# S95. PREVALENCE AND CLINICAL CORRELATES OF COMORBID OBSESSIVE-COMPULSIVE DISORDER IN PATIENTS WITH SCHIZOPHRENIA

**DOI:** 10.1093/schbul/sby018.882

**Published:** 2018-04-01

**Authors:** Lee Bora, Kyung Sue Hong, Ji Hyun Baek, So Yung Yang

**Affiliations:** 1 Samsung Medical Center; 2 Sungkyunkwan University School of Medicine, Samsung Medical Center

## Abstract

**Background:**

Obsessive compulsive symptoms (OCS) commonly occur in the course of schizophrenia. However, reported rates of comorbid obsessive compulsive disorder (OCD) in schizophrenia were highly variable among studies. In addition, influences of OCS on the symptomatology and functioning of schizophrenia have not been fully explored. The aim of this study was to investigate the clinic-based prevalence rate of OCD in schizophrenia patients, and to evaluated clinical correlates of the comorbidity.

**Methods:**

Patients with schizophrenia (n=320) were recruited and lifetime clinical characteristics were evaluated comprehensively. Patients having comorbid OCD (OCD group, n=66) and those without OCD (the non-OCD group, n=254) were compared in terms of clinical characteristics and cognitive functioning.

**Results:**

OCD was found in 20.6% of the subjects. Earlier age at onset, male gender, and higher level of education were associated with comorbid OCD. In terms of individual symptoms and symptom dimensions, ‘anxiety (p=0.009) and ‘depression (p=0.001)’ were more frequently observed in the OCD group than in the non-OCD group. The prodromal impairment was higher in the non-OCD group (p=0.016). The OCD group showed better performance in working memory domain (p= 0.003), and other cognitive domains did not show any significant group difference.

**Discussion:**

The prevalence rate of OCD in the current subjects was within the range of previously reported comorbidity rates in schizophrenia patients from other populations. Association of OCS with anxiety and depressive symptoms seems to be a common finding which was also reported in previous studies of schizophrenia and bipolar disorders. Regarding cognitive functions, inconsistent results including the current report have been generated suggesting heterogeneous developmental mechanisms of OCS in schizophrenia.

